# T cells heal bone fractures with help from the gut microbiome

**DOI:** 10.1172/JCI167311

**Published:** 2023-04-17

**Authors:** Rajeev Aurora, Matthew J. Silva

**Affiliations:** 1 Department of Molecular Microbiology and Immunology, Saint Louis University School of Medicine, St. Louis, Missouri, USA.; 2 Department of Orthopedics, Washington University School of Medicine, St. Louis, Missouri, USA.

## Abstract

Immune cells play an important functional role in bone fracture healing. Fracture repair is a well-choreographed process that takes approximately 21 days in healthy mice. While the process is complex, conceptually it can be divided into four overlapping stages: inflammation, cartilaginous callus formation, bony callus formation, and remodeling. T cells play a key role in both the cartilaginous and bony callus phases by producing IL-17A. In this issue of the *JCI*, Dar et al. showed that T cells were recruited from the gut, where the gut microbiota determined the pool of T cells that expressed IL-17A. Treatment with antibiotics and dysbiosis reduced the expansion of IL-17–expressing CD4^+^ T cells (Th17) and impaired callus formation. These findings demonstrate crosstalk among the gut microbiota, the adaptive immune system, and bone that has clinical implications for fracture healing.

## Stages of fracture healing

A fracture initiates a complex cascade of molecular, cellular, and tissue events that typically leads to bone healing that is dependent on an appropriately stable mechanical environment. Work by numerous authors has led to a good understanding of the basic science of fracture healing (1–5). Fractures that are stable and have minimal displacement of the broken surfaces, such as stress fractures, can heal by primary (also called intramembranous) bone formation. More commonly, the bone surfaces are displaced, and there is some motion at the fracture site, which favors secondary healing via the process of endochondral ossification. This process has been broadly characterized by temporal progression through four overlapping stages: inflammation, cartilage callus formation, bone callus formation, and remodeling. A failure to progress through any one of these stages can result in delayed healing or nonunion. While these stages provide a helpful conceptual framework, it is recognized that the pace of events within and between stages varies spatially and that aspects of each stage may be present together within a fracture callus. In this issue of the *JCI*, Dar et al. primarily examined immune cell and cytokine responses at day three, corresponding to the initial inflammatory phase, followed by functional outcomes at the end of bony callus formation at days 14 and 21 and remodeling at day 35 (6).

## Immune cells and cytokines in fracture healing

Inflammation is a hallmark of the early response to fracture, like injury in other tissues (4). Successful healing depends on the initiation of a robust inflammatory response, which occurs over hours to days, as well as timely resolution of this response that takes place within days to week and involves many cell types (1, 2, 4). Fracture disrupts blood vessels, which leads to the formation of a hematoma, known as a fibrin clot, which in turn acts as a scaffold for immune cells and cytokines (3, 7). The events that modulate the initial recruitment of cells are not fully understood, but tissue-resident macrophages and other local cells are increasingly recognized as important in detecting damage and initiating a response (4). Specifically, bone-resident macrophages, termed “osteomacs,” have been shown to play a role in osteogenesis during bone repair healing (8, 9). Neutrophils are recruited within 24 hours and are the predominant leukocyte within the hematoma, where they phagocytose pathogens and debris. By 24 to 48 hours, there is a large influx of monocytes and proinflammatory (M1) macrophages; these and other cells produce numerous cytokines including IL-1, IL-6, TNF-α, and CCL2 (4, 5). Neutrophils, in part, regulate the macrophage response, as evidenced by an increase in macrophages at the site of isolated femur fractures within a week in mice exposed to neutrophil-reducing antibodies (10). Importantly, these early inflammatory changes lead to impaired functional healing, as measured by reduced callus bone volume and mechanical properties at day 21 (10).

Macrophages are central to fracture healing, both as phagocytes that clear tissue debris and apoptotic cells, and as modulators of inflammation and the anabolic phases of healing related to cartilage and bone formation (5, 11, 12). Broadly, a switch of macrophages from the M1, proinflammatory phenotype early to the M2, regenerative, phenotype later appears to be essential for the resolution of inflammation and successful fracture healing (4, 12). Inflammatory macrophages predominate in the granulation tissue that forms in the first seven days after a fracture and precedes the formation of cartilage (11). Macrophage depletion (in MaFIA-transgenic mice), initiated either at the time of fracture or five days later, results in failure to form the granulation tissue and subsequent cartilage callus (11). Similarly, macrophage depletion starting at the time of fracture blunts cartilage formation and delays endochondral ossification, leading to inferior mechanical properties (13). Conversely, immunomodulatory treatments can enhance healing. Administration of the pro-macrophage cytokine CSF-1 seven days after fracture boosted macrophage numbers and the size of the cartilage callus (11), while administration of IL-4 and IL-13 boosted M2 macrophages and led to improved healing (13).

T cells are present in the fracture hematoma, and different subsets of T cells may impair or promote bone healing (3). Recently, Ono et al. reported that γδ T cells are present at the site of bone injury and that IL-17A from these cells promotes bone formation in the defect (14). Dar et al. extended this work to focus on the effects and origins of T cells, both αβ and γδ subtypes, in a more clinically relevant model of full fracture that heals via endochondral ossification (6). αβ T cells are activated via T cell receptor (TCR) binding to antigen in the context of the MHC of antigen-presenting cells (APCs). In contrast, γδ T cells are activated by the cytokines IL-1β, IL-18, and IL-23, which are produced by APCs in the absence of MHC-TCR recognition. Thus, γδ T cells amplify the response of αβ T cells.

## Gut microbiome and the immune system

The immune system is particularly connected with gut microbiota (15). The small and large intestines form a long mucosal tract where food is digested and the nutrients are absorbed and therefore needs to be permeable. Since ingested food is not sterile and the gastrointestinal (GI) tract is open, it is teeming with bacteria and fungi. Moreover, some bacteria are symbiotic because in return for a steady food supply they produce metabolic products that are essential nutrients not readily available in the host’s diet (16). As microbes are both unavoidable and essential, the species present and their abundance must be regulated, as overgrowth is pathogenic. The immune system plays a critical role in recognizing and eliminating pathogens and maintaining the abundance of the commensals and symbionts (15). It is notable that some species are tolerated in the gut. For instance, a common gut commensal, *Escherichia coli*, does not invoke an immune response in the gut. The same strain of *E*. *coli* transferred to the bladder via a contaminated catheter, or to the lung by intubation, evokes a strong immune response. T cells are particularly important for maintaining both tolerance to *E*. *coli* in the gut and an inflammatory response at other mucosal surfaces, suggesting that there is a T cell memory of microbial species that is tissue specific. This result also indicates that the microbiome educates T cells in a tissue-specific manner (17). Moreover, these observations also suggest that a majority of T cells take up long-term residence in the gut (17). Changes to the microbiota, such as by fecal transplantation or antibiotic treatment, lead initially to the formation of proinflammatory T cells in some instances, and then most likely to the formation of long-term memory T cells.

Dar et al. introduced segmented filamentous bacteria (SFB) into mice. SFB are a host-adapted genetic cousin of the genus *Clostridium* that bind to absorptive epithelium cells (6). While SFB are commensals, they, uniquely, induce a response from IL-17–expressing CD4^+^ T cells (Th17) when introduced into a naive host. Notably, IL-23 produced by DCs promotes Th17 responses, as it activates γδ T cells (18, 19).

## Gut-resident T cells are recruited in bone fracture

**Figure 1:**
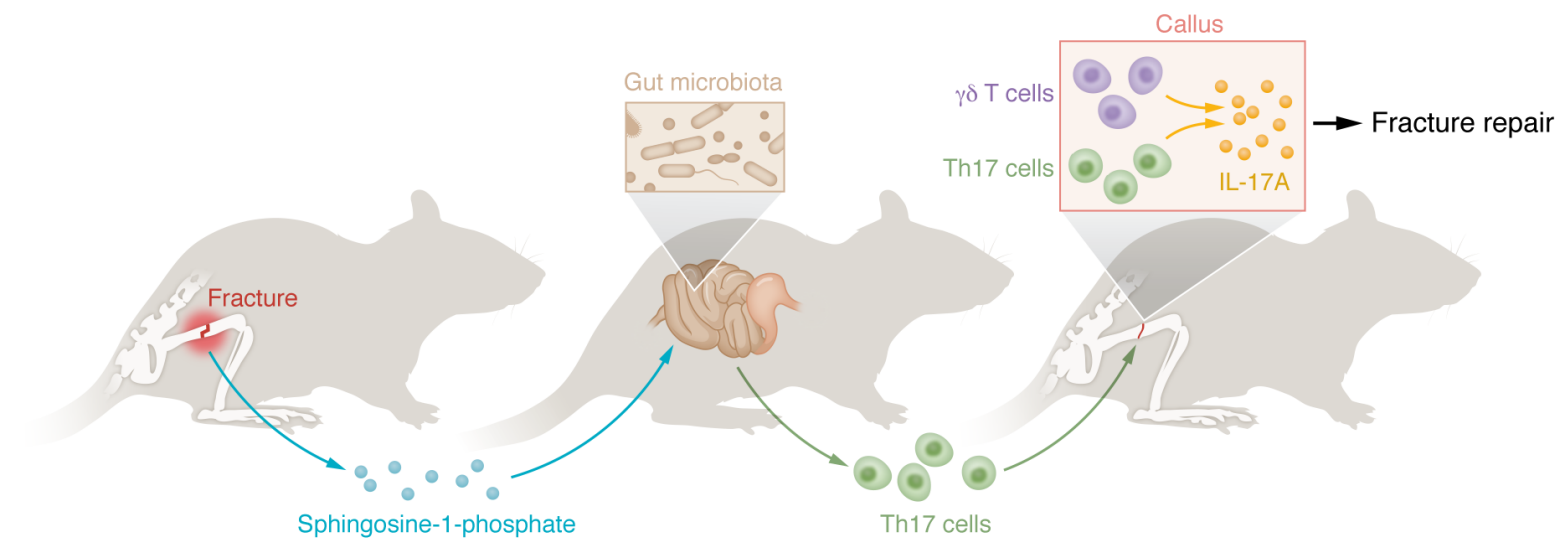
T cell subsets have a regulatory role in the inflammatory phase of fracture healing. The findings of Dar et al. (6) show that bone fractures recruit gut-resident T cells. The strength of the inflammatory response by T cells determines the quality of callus formation and biomechanical strength. Bone fractures result in the release of sphingosine-1-phosphate. The gut microbiota determines the pool and expansion of Th17 cells. Th17 cells migrate to the callus. Callus γδ T cells and recruited Th17 cells contribute to callus formation and the anabolic stage of fracture repair.

A role for the gut microbiome as a regulator of skeletal biology has emerged in the past several years (20, 21). For example, gut microbiota can affect bone loss following ovariectomy in a model of postmenopausal osteoporosis (22) and also influence the anabolic effect of the FDA-approved parathyroid hormone used to treat osteoporosis (23). Dar et al. extend this work and propose a model whereby the gut microbiome regulated, in part, the inflammatory phase of fracture healing (Figure 1) (6). Bone fractures release sphingosine-1-phosphate (S1P) to recruit T cells. S1P, typically produced by endothelial cells, acts as a coupling factor between osteoclasts, which are cells that resorb bone, and osteoblasts, which produce new bone (24). S1P recruits macrophages and T cells (25). The authors show that blockade of S1P reduced T cells in the callus and that altering the gut microbiome by transplanting SFB increased Th17 cells in the callus. Furthermore, by tracking a fraction of these T cells, marked in the Peyer’s patches by exposure to UV light in reporter mice, they demonstrated that gut-resident T cells were recruited to the callus. Finally, inhibition of the recruitment of T cells led to a delay in bone callus formation at day 14, and the repaired bone had reduced biomechanical properties at day 35. These results confirm that the repair process is serialized and that events in early stages of healing delay or prevent later stages and affect the functional quality of the repaired bone (6).

## Clinical implications and future directions

There are 12 to 15 million fractures in the US each year, leading to more than 18 million health care visits (26). These injuries result in 60 million workdays lost, more than twice the number for heart disease and stroke combined (27). While bones have a remarkable ability to heal via scarless regeneration, the overall rate of fracture nonunion is approximately 5%, with rates greater than 10% for weight-bearing long bones, such as the tibia and femur (28). Thus, there is a need to identify interventions that can improve fracture healing. One of the clinical implications of the study by Dar et al., (6) as pointed out by the authors, involves optimizing the microbiome by restricting the use of broad-spectrum antibiotics and potentially providing probiotics immediately after bone fracture. Antibiotics may lead to dysbiosis and delay the repair process. Promoting Th17 responses may also promote repair. While a bone can break occur at any age, there is higher risk of bone fractures, especially fragility fractures, in aged and osteoporotic individuals. Furthermore, in this population, fracture healing is slowed and fails in some individuals. Diabetes can also delay callus formation. It will be important in future studies to understand whether and how the gut microbiome influences the acute inflammation induced by bone fractures in settings of chronic inflammation in diabetes, aging, and the postmenopausal state. Last, how T cells move from the gut to the bone is another area for future study. T cells can travel between tissue via blood or lymphatic vessels. It has been suspected for a long time that bone contains lymphatic vessels, but definitive evidence has been lacking (29). A recent study provided direct evidence for lymphatic vessels (30). The vasculature also plays a key role in regulating bone homeostasis (31, 32). Future studies will likely further elucidate the interactions among microbiota, T cells, lymphatic tissue, and the vasculature in fracture healing.

## Version 1 04/17/2023

Electronic publication
